# Supplementary material for: Modelling Skylarks (Alauda arvensis) to Predict Impacts of Changes in Land Management and Policy: Development and Testing of an Agent-Based Model
Source: PLoS One. 2013 Jun 6;8(6):e65803. doi: 10.1371/journal.pone.0065803 (PMC3675089; doi:10.1371/journal.pone.0065803)
Supplement: Supporting Information S4 — The skylark ODdox as a zipped archive. (ZIP) [file pone.0065803.s004.zip › Skylark_ODdox/rastermap_8cpp.html]

ALMaSS Skylark ODdox: rastermap.cpp File Reference


ALMaSS Skylark ODdox
 2.0


- Main Page
- Related Pages
- Classes
- Files

- File List
- File Members

Macros |
Variables

rastermap.cpp File Reference

`#include <cstdio>`  
`#include <stdlib.h>`  
`#include "maperrormsg.h"`  
`#include "rastermap.h"`  
`#include "configurator.h"`

|  |  |
| --- | --- |
| Macros | |
| #define | \_CRT\_SECURE\_NO\_DEPRECATE |

|  |  |
| --- | --- |
| Variables | |
| CfgBool | cfg\_manipulation1\_on ("RASTER\_MANIPULATION\_ONE\_ON", CFG\_CUSTOM, false) |
| CfgInt | cfg\_rastermapmanipmultiply ("RASTER\_MANIPULATION\_MULTIPLYFACTOR", CFG\_CUSTOM, 2) |

---

## Macro Definition Documentation

#define \_CRT\_SECURE\_NO\_DEPRECATE

---

## Variable Documentation

CfgBool cfg\_manipulation1\_on("RASTER\_MANIPULATION\_ONE\_ON", CFG\_CUSTOM, false)

Referenced by RasterMap::RasterMap().

CfgInt cfg\_rastermapmanipmultiply("RASTER\_MANIPULATION\_MULTIPLYFACTOR", CFG\_CUSTOM, 2)

Referenced by RasterMap::Manipulation1().


- CJT
- MSVC
- ALMaSS Working Source
- Landscape
- rastermap.cpp
- Generated on Thu Jan 10 2013 13:15:35 for ALMaSS Skylark ODdox by
   1.8.1.1
